# Training wheels needed: Lessons in professionalism from a liberal deferral policy

**DOI:** 10.1007/s40037-019-0520-7

**Published:** 2019-06-04

**Authors:** Michelle Daniel, Tamara Gay, Rajesh Mangrulkar, Paula Ross, Sara Weir, Emily Hogikyan, Owen Thompson, Sally Santen

**Affiliations:** Medical School, University of Michigan, Ann Arbor, MI USA

**Keywords:** Professionalism, Attendance, Self-regulation, Wellness

## Abstract

Learning to self-regulate is an important aspect of professionalism. Thus, in 2015–16, the University of Michigan implemented a learner-centred ‘deferral’ policy called ‘trust and track’ in the preclinical phase. This gave students the autonomy to decide whether to attend required experiences, take quizzes and exams on schedule, or submit assignments on time. Surprisingly, quiz and exam deferrals remained relatively stable, but required experience deferrals more than doubled. While late assignments were not specifically tracked, there were multiple reports of assignments being months overdue. Some reasons for deferrals exhibited questionable judgement. Behavioural patterns carried forward, with an unusual spike in deferrals of licensure exams and requests for time off in the clinical phase. Wellness indices did not improve, despite learners having more autonomy and flexibility. It became clear to us that novice learners need clear professional expectations with limits to assist in developing professional behaviours. In 2016–17, we implemented a stricter policy that set clear expectations, established limits, and provided guidance on acceptable reasons to defer. We simultaneously implemented other measures to promote wellness. The moral of the story is that ‘training wheels’ are needed to help early learners develop the professional behaviours expected of practising physicians.

**Electronic supplementary material:**

The online version of this article (10.1007/s40037-019-0520-7) contains supplementary material, which is available to authorized users.

## The story

Professionalism is fundamental to the practice of medicine [1]. Self-regulation is one component of professionalism, and thus creating an environment that fosters the development of self-regulation is important [2]. At the University of Michigan, we believe medical students are responsible, adult learners. We have long embraced our learners’ capacity to self-regulate through optional lecture attendance and flexible testing (taking quizzes and exams on weekends in un-proctored, campus-based settings, governed by the honour code.) Thus, when our counsellors expressed they were over-burdened with tracking and granting permission for students to miss required experiences, quizzes and exams, we decided to implement a more student-centred “deferral” policy, in keeping with our other professional development practices.

In this new model, dubbed “trust and track”, we gave students the autonomy to self-regulate, encouraging them to balance their professional obligations with decisions that fostered their personal wellness and prevented burnout. We believed this process modelled clinical clerkships, residency and practice in that trainees and physicians have to balance learning, work and life priorities. In “trust and track”, students were allowed to decide whether to 1) attend required curricular experiences, 2) take quizzes and exams as scheduled, or 3) submit assignments on time. There were no limits on the number of deferrals and no repercussions (beyond the risk of falling behind) for students who deferred. Students had to complete a make-up assignment if they deferred a required experience (though there were no deadlines for completion). For quizzes and exams, students typically had to take the test within one week, but longer extensions could be negotiated with their counsellor. To obtain credit, assignments ultimately had to be turned in, but there was no penalty for being late. (For comparison with other curricula, our students spend an average of 8.5 h in required activities, complete one assignment, and have a quiz or exam weekly).

The web-based, self-report system was implemented in 2015–16 for the first year (M1) and second year (M2) classes in our Pass/Fail preclinical curriculum. Students only had to log in to the system, report the activity, quiz or exam they would be deferring and provide the reason for the deferral. While there was a list of common acceptable reasons for guidance, students could also list other reasons. If the deferral was for a quiz or exam, the counsellor was notified for awareness. We monitored utilization and for changes in wellness indices.

## Surprising outcomes

Quiz and exam deferrals in the 2015–16 M1 cohort remained relatively stable compared with 2014–15, yet deferrals for required experiences more than doubled (Tab. 1). There was a 30% increase in required experiences during this time frame due to a transition to a curriculum with more active learning experiences, including a longitudinal clinical skills course, but the deferral use seemed out of proportion to this change. The numbers of deferrals for required experiences in the M2 class increased even more than for M1s, yet the number of required experiences in that cohort did not change. While the majority of reasons for deferrals were acceptable, some exhibited more questionable judgement (e.g. travel to undergraduate alma mater for a rivalry football game, homecoming alumni activities, date night.) Furthermore, we noticed some concerning patterns in deferral utilization, with most students having 0–1 deferrals while a few had 15–20. We also observed a large number of deferrals for “shadowing”. While shadowing practising health professionals is educational, in the self-regulated model students did not feel empowered to negotiate an alternate time for these activities, even when it was feasible. We did not track late assignments specifically, but there were many anecdotal reports of students not submitting required assignments for months after the due date.

**Table 1:** M1 deferrals according to system and academic year

	Counsellor regulated 2014–15^b^	Student (self-)regulated 2015–16	Difference between 2014–15 and 2015–16
Number of students	176	170	−6
Weekly quiz/exam deferrals	422	398	−24
Required experience^a^ deferrals	250	591	341
Total number of deferrals	672	989	317
Average # deferrals/student	3.9	5.6	1.7

^a^ *There was a 30% increase in required experiences in 2015–16 compared with 2014–15*

^b^ *The accuracy of the number of deferrals for 2014–15 were limited due to a less formal tracking procedure*

We observed some additional concerning and unintended consequences. Twenty-four M2 students (~14% of the class) deferred the United States Medical Licensing Examination (USMLE) Step 1 by a month or more, affecting their entry into core clerkships per our policies, with potential downstream repercussions of delayed graduation. While each year we have a handful of students who delay Step 1, these numbers were beyond what we had ever seen. We hypothesized that students were not accustomed to managing their time and strictly adhering to an exam schedule, so when the stakes were high, some panicked or simply fell back on their usual practice: If they didn’t feel ready, they deferred. For students who struggle with test anxiety, deferrals may generate a perpetual cycle of delay. When the students from the self-regulated deferral policy moved into the clinical environment, administrators and clerkship directors complained about receiving a record number of requests for days off, impacting the healthcare team and potentially, patient care.

When we (the administration) expressed concern at the rising numbers of deferrals (and some of the stated reasons), students responded that we failed to set clear expectations for their behaviour. Through class meetings and discussions with curricular representatives, it became clear that students viewed their decisions as “individual” in the preclinical years, affecting no one but themselves. They wanted more guidance, yet, they did not see a policy that set limits as “scaffolding” the professional choices they would need to make later in training. Many viewed their preclinical studies as a last opportunity for flexibility before others were relying on them. They also firmly believed that decisions made in the preclinical years were in no way predictive of their future behaviour as clinical students or practising physicians. While correlation does not mean causation, the evidence suggested that learners struggled to change established patterns of behaviour once they entered the clinical environment.

Interestingly, of the wellness indices we tracked, wellbeing index [3] and resiliency [4], we did not observe significant improvement in the student-regulated model, compared with the historical counsellor-regulated model (Tab. 2). We had expected the more liberal deferral policy to enhance wellness, as autonomy and flexibility were repeatedly emphasized by students as factors influencing their wellbeing.

**Table 2:** End of M1 survey on well-being resiliency, and perceived stress

	Counsellor regulated 2015			Student (self-)regulated 2016			Independent t‑test 2015 vs 2016	
Scales	Mean	SD	*N*	Mean	SD	*N*	Mean diff	*p*-value
*Well-Being Index*	4.28	4.61	146	4.77	4.71	144	0.48	0.378
*Resiliency*	8.53	1.17	146	8.38	1.28	144	−0.15	0.291

On both scales, higher numbers represent worsening of well-being, resiliency and perceived stress.

When *p* < 0.05, differences may be considered statistically significant

## Lessons learned

Students need clear professional expectations with limits and scaffolding to assist in developing professional behaviours [5], especially since students enter the clinical environment earlier than ever before. A recent study showed that three or more absences from attendance-required sessions in the preclinical years predicted unprofessional conduct during the clinical years (odds ratio 4.47) [6]. An earlier study showed connections between behavioural issues during medical school and reports brought before medical licensing boards [7, 8]. Our findings suggest that behaviours may indeed carry forward. While many students can self-regulate upon arrival to medical school, some students require specific expectations and limits to help them make choices. We changed our deferral policy for 2016–17 with student input. The new policy set clear expectations concerning the number of deferrals allowed per year for required experiences, quizzes and exams and provided guidance on acceptable reasons for deferring (e.g. illness, major family/close friend event or emergency, significant religious holiday, or to attend or present at a conference). The new policy also permitted three “free” late assignments with one week to make them up and outlined specific consequences of going over the pre-determined limits. When creating the new, more stringent attendance policy, we implemented other measures to enhance wellness. These included timelier provision of schedules, seven scheduled quiz-free weekends, quiz/exam extensions for major student life events affecting the bulk of the student body, and clustering of required experiences mid-week so that students could stream content Mondays and Friday afternoons.

We operationalized a small deferral review committee consisting of representation from student affairs and curriculum to review students who exceeded the deferral limits. While 23 students in the 2016–17 cohort exceeded the “limits,” the majority of students used the deferrals for acceptable reasons, requiring no further action. Only one instance required the filing of a professionalism concern note to our competency committee (for attending a concert despite their deferral request having been denied).

We observed a marked decrease in deferral utilization in 2016–17 (*n* = 172 M1 students) under the new policy with only 71 deferrals for quizzes (82% reduction), 263 deferrals for required experiences (55% reduction), and 334 total deferrals (66% reduction). We saw a slight, but significant worsening in the well-being index (mean 5.84, *p* = 0.06) despite the afore-mentioned measures to enhance wellness. The resiliency index was largely unchanged (mean 8.31, *p* = 0.617). We maintained the new deferral policy for 2017–18, but increased the number of deferrals allowed for quizzes and exams by one. This small change was made based on student and counsellor feedback concerning the “stress” associated with quiz and exam deferrals, particularly for struggling students.

## Moral of the story

In retrospect, our self-regulated deferral policy was an “epic failure”. We learned that “training wheels” are required to help early learners develop the professional behaviours we expect from practising physicians. Educators and learners must work together to find the “middle ground”, that balances student wellness and autonomy, while fostering the development of professional behaviour.

A liberal deferral policy seems desirable to empower students to make professional decisions, to reduce the burden on counsellors and to improve students’ wellness. However, as educators, we are ultimately responsible for providing strong guidance surrounding professional development.

## Caption Electronic Supplementary Material

Professional Expectations for the Scientific Trunk Matriculating Class of 2016. This supplement provides the full written deferral policy implemented in 2016–17. The policy sets clear expectations and provides guidance for appropriate deferral usage.
