# Supplementary material for: Training wheels needed: Lessons in professionalism from a liberal deferral policy
Source: Perspect Med Educ. 2019 Jun 4;8(3):187–90. doi: 10.1007/s40037-019-0520-7 (PMC6565661; doi:10.1007/s40037-019-0520-7)
Supplement: Supplementary file 1 — Professional Expectations for the Scientific Trunk Matriculating Class of 2016. This supplement provides the full written deferral policy implemented in 2016–17. The policy sets clear expectations and provides guidance for appropriate deferral usage. [file 40037_2019_520_MOESM1_ESM.docx]

| **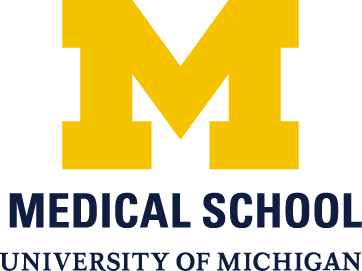** | **Professional Expectations for the Scientific Trunk**  **Matriculating Class of 2016**  (effective 7/15/16) |
| --- | --- |

**Preamble**

We understand that our students value personal and professional experiences outside of school. From the curriculum side, we are doing what we can to cluster required experiences and we are providing 6 quiz free weekends in the 2016-2017 Scientific Trunk. We have made every attempt to schedule around major events in which a significant part of the student body participates. There will be no quiz the weekend of Tag Days. The weekend of Biorhythms the quiz will remain open for everyone until Monday at 10 PM. The weekend of the Smoker the exam will remain open for everyone until Tuesday at 10 PM. Specifics will be detailed on the Canvas calendar.

**Required Experiences / Quizzes and Exams**

*The expectation is that students will attend all required experiences and take quizzes and exams on time.*

Students may miss a predetermined number (see below) of required experiences / quizzes / exams for a short list of acceptable reasons [illness, major family/close friend event or emergency (i.e. wedding, funeral), significant religious holiday, attending^[[1]](#footnote-1)^ or presenting at a conference, participation in Office of Medical Student Education (OMSE) committees or other formal medical school functions]. The numbers of deferrals for each course were chosen based on rates of utilization in previous years. They are meant to provide guidance when planning activities to ensure students stay on track and thrive in medical school. Saving deferrals for illness / emergencies is *not necessary* as students may exceed the predetermined numbers and still be using the deferral system appropriately assuming that 1) communication is timely and appropriate, 2) the reasons are acceptable, 3) the student is otherwise meeting professional expectations, and 4) no concerning patterns arise.

**The Procedure:** The **course administrator** will be informed directly by the student (via email with a copy to the house counselor) if they will be missing a required experience, and this will be tracked at the course level. If a student believes they have a compelling reason (other than those listed above) to miss a required experience, they should discuss this with their counselor ahead of time. If the counselor agrees, the student should then email the course administrator (cc’ing the counselor) that they have an acceptable absence. The course administrator will reply to the student and direct them to the Canvas page with the remediation information including the due date of the assignment. Quiz / exam deferrals **must** be approved by the **counselor**, and the counselor will be responsible for tracking these.

Please use the following email templates to request a required experience / quiz / exam deferral:

**Required Experience Deferral Email (to the course admin, cc’ing counselor)**

Subject: Deferral Request: Course, Experience Title/Date (ex: Deferral Request: Foundations, Ethics Small Group, 9/12/16)

Body of the email: Indicate which of the acceptable reasons for which you will be deferring or confirm that another reason has been discussed and approved by the house counselor. Students will receive a confirmation email when the request for deferral has been approved.

**Quiz or Exam Deferral Email (to the counselor)**

Subject: Deferral Request: Course, Quiz / exam / lab practical or both, open date of the quiz exam (ex. Deferral Request: Renal, Quiz #1, 12/9/16).

Body of the email: Indicate which of the acceptable reasons for which you will be deferring. If there is another compelling reason for your request, please provide a detailed description of the circumstance. The counselor will review your request and reply to you in a timely manner. Deferrals that are approved, but not actually used, do not “count” against a student’s total number of deferrals. Be sure to let your counselor know if an approved deferral is not used, so that your record can be corrected.

**NB:** Planned deferrals should be requested as far in advance as possible but must be requested at least one (1) week in advance. Emergent deferrals should be submitted as soon as possible and will be automatically approved if the course administrator or house counselor is notified by 12:00 noon the day after the required experience or quiz / exam. If these request guidelines are not met, students will not be allowed to make up the experience, take the assessment or receive credit. In the event of illness, students may be required to seek medical attention and bring verification. Being unprepared is not considered an acceptable reason to defer, although extenuating circumstances are always considered on an individual basis.

Each course will lay out a clear attendance policy at the beginning of the semester in their Canvas page and course orientation. For each course, the following number of deferrals is acceptable:

| **Course** | **Number of Deferrals**  **August 2016 – September 2017** |
| --- | --- |
| All Trunk Organ System Courses + Chief Concern Course  (Course Administrator: XXX \| Email: XXX) | Two (2) combined |
| Doctoring  (Course Administrator: XXX \| Email: XXX) | Two (2) |
| Pathway Interdisciplinary Seminars + Leadership  (Course Administrator: XXX \| Email: XXX) | One (1) combined |
| Initial Clinical Experience  (Course Administrator: XXX \| Email: XXX) | One (1) |
| Quizzes + Exams  (Contact: House Counselor  Salk: Counselor XXX, Email: XXX  Sanford: Counselor XXX, Email: XXX  Fitzbutler: Counselor XXX, Email: XXX  Hamilton: Counselor XXX, Email: XXX | Two (2) |

Please note, if students request a deferral for a full day, and if there is more than one required experience that day in the same course (i.e. patient presentation and a small group in Foundations), this will only count as one deferral. If students are out for a full day and this affects two different courses, the deferral will count as a deferral for both courses (i.e. Foundations + Doctoring), and students will need to request deferrals from both course administrators.

When required sessions are deferred, remediation assignments are due within one week of the missed experience. Remediation assignments should be sent to the course/sequence director and course administrator, unless otherwise specified on Canvas.

Missed required experiences beyond these numbers will trigger the following:

1. A conversation/meeting with the house counselor or the Assistant Dean for Student Services (for quizzes/exams)
2. Review by the deferral committee (comprised of the Assistant Deans for Student Affairs and Curriculum, and the house counselors) and reporting to Competency Committee for possible action. The deferral committee evaluates the use of deferrals to determine if students are appropriately meeting professional expectations. They make recommendations concerning whether or not a student needs discussion at Competency Committee based on the following criteria: Was communication timely and appropriate? Were the reasons acceptable? Is the student otherwise meeting professional expectations? Are there any concerning patterns? Students who will be discussed at competency committee will be informed by the house counselors and have the opportunity to write a letter of explanation to the board.

**Assignments**

*The expectation is that all assignments will be turned in on time.*

We understand that students sometimes have conflicting priorities; therefore up to **three (3) late assignments** are permissible across all courses. For these late assignments, students are responsible for turning them in within one (1) week. If the Canvas tracking system shows that a student has more than three (3) total late assignments OR if any assignment is > one (1) week late, there will be automatic reporting to the deferral committee and Competency Committee for discussion of whether a student is appropriately meeting professional expectations for possible action. If there is a valid reason for an assignment to be late (i.e. a clinic visit cannot be scheduled in a timely fashion with your Doctoring family), *a new deadline* must be agreed upon with the course administrator / leader in advance of the due date, that will be entered into Canvas. Changing the due date will not be tracked as a late assignment, but it will count as a late assignment if a student misses the renegotiated deadline.

As per course grading guidelines, lack of completion of assignments may also be cause to assign a student an Incomplete (I) for a course with subsequent referral to the Competency Committee.

**Once students enter clinical rotations, the specifics of this document no longer apply.**

1. Per policy, students may only receive OMSE funding when presenting at a conference. [↑](#footnote-ref-1)
